# Healthcare professionals' and consumers' knowledge, attitudes, perspectives, and education needs in oncology pharmacogenomics: A systematic review

**DOI:** 10.1111/cts.13672

**Published:** 2023-11-22

**Authors:** Chiao Xin Lim, Alistair Bozkurt, Zi Yue Chen, Abbey Hird, Joanne Wickens, Smaro Lazarakis, Safeera Y. Hussainy, Marliese Alexander

**Affiliations:** ^1^ Pharmacy, School of Health and Biomedical Sciences RMIT University Bundoora Victoria Australia; ^2^ Health Sciences Library, Royal Melbourne Hospital Parkville Victoria Australia; ^3^ Pharmacy Department, Peter MacCallum Cancer Centre Melbourne Victoria Australia; ^4^ Sir Peter MacCallum Department of Oncology The University of Melbourne Melbourne Victoria Australia; ^5^ Department of General Practice, School of Public Health and Preventive Medicine Monash University Clayton Victoria Australia

## Abstract

Clinical implementation of pharmacogenomic (PGx)‐guided prescribing in oncology lags behind research evidence generation. We aimed to identify healthcare professionals' (HCPs) and consumers' knowledge, attitudes, perspectives, and education needs to inform strategies for implementation of scalable and sustainable oncology PGx programs. Systematic review of original articles indexed in EMBASE, EMCARE, MEDLINE, and PsycInfo from January 2012 until June 2022, following Preferred Reporting Items for Systematic Reviews and Meta‐Analyses (PRISMA) guidelines and using the Mixed Methods Appraisal Tool. PROSPERO registration number CRD42022352348. Of 1442 identified studies; 23 met inclusion criteria with 87% assessed high quality. Of these, 52% reported on HCPs, 35% on consumers, and 13% on both HCPs and consumers. Most were conducted in the United States (70%) and included multiple cancer types (74%). Across studies, HCPs and consumers mostly perceived value in PGx, however, both groups reported barriers to utilization, including cost, lack of consistent recommendations across guidelines, and limited knowledge among HCPs; test accuracy, clear testing benefits, and genomic information confidentiality among consumers. HCPs and consumers value and want to engage in PGx strategies in oncology care, however, are inhibited by unmet needs and practice and knowledge gaps. Implementation strategies aimed at addressing these issues may best support increased PGx uptake in oncology practice.

**Study Highlights**

**WHAT IS THE CURRENT KNOWLEDGE ON THE TOPIC?**

A significant number of medicines utilized for the treatment and supportive care of individuals with cancer have pharmacogenomic (PGx) recommendations outlined in international PGx clinical guidelines. However, clinical implementation of PGx‐guided prescribing in oncology still lags behind research evidence and guideline publications.

**WHAT QUESTION DID THIS STUDY ADDRESS?**

What are oncology healthcare professionals (HCPs) and consumers knowledge, attitudes, perspectives, and education needs for PGx?

**WHAT DOES THIS STUDY ADD TO OUR KNOWLEDGE?**

Both HCPs and consumers appreciated the value of PGx in personalized medicine and want to engage in PGx strategies in oncology care, but are inhibited by barriers to utilization, including cost, lack of consistent recommendations across clinical guidelines, and limited knowledge among HCPs; test accuracy, clear testing benefits, and genomic information confidentiality among consumers.

**HOW MIGHT THIS CHANGE CLINICAL PHARMACOLOGY OR TRANSLATIONAL SCIENCE?**

Implementation strategies, including education and consistency of recommendations across evidence‐based clinical guidelines aimed at addressing practice and knowledge gaps are key to supporting routine PGx screening in practice.

## INTRODUCTION

Most individuals diagnosed with cancer will require treatments including multiple and high‐risk medicines with common occurrence of unplanned emergency presentations and hospital admissions, consequent to adverse medicines events and symptom‐driven complaints.^1^ Events are often linked to individual variations in response and toxicity within standard dosing limits and a general “one size fits all” approach. Within a population, substantial genetic variations may exist, which may result in unpredictable outcomes of treatment with pharmacological agents.^2^ Pharmacogenetics or pharmacogenomics (PGx) is the study of interactions between genes and medicines.^3^ These terms are often used interchangeably – pharmacogenetics refers to the study of variations in one or several genes as related to drug response, whereas pharmacogenomics refers to the study of variations in genome as related to drug response.^3^, ^4^ We have chosen to use pharmacogenomics as it is a broader term and pharmacogenomic testing and research are becoming more common as a result of advances in genomic sequencing technology. PGx testing, which involves the detection of genomic variations underlying differences in interindividual drug metabolizing enzymes, receptors, and drug targets, has instigated a new paradigm of personalized medicine, as it allows clinicians to predict patients' response to specific treatments based on their genetic variations.^5^

PGx‐guided prescribing has been touted as the future of medicine, as treatment selection and dosage adjustment can be informed by patients' genetic data to ensure treatment efficacy and minimize toxicity, leading to improved health outcomes for patients.^6^, ^7^ However, uptake of PGx‐guided prescribing remains highly variable across health services globally.^8^ Clinical implementation of PGx‐guided prescribing remains challenging due to regional implementation requirements and administrative burden to manage these requirements.^9^

A number of medicines utilized for the treatment and supportive care of individuals with cancer have PGx recommendations outlined in international PGx clinical guidelines, including the Clinical Pharmacogenetics Implementation Consortium (CPIC) and the Dutch Pharmacogenetics Working Group (DPWG).^10^, ^11^, ^12^ Out of 26 drug‐gene pairs published in CPIC guidelines, three are oncology‐specific, including *CYP2D6*‐Tamofixen, *DPYD*‐fluoropyrimidines, and *TPMT*/*NUDT15*‐thiopurines; and others relevant to pain and supportive care medicines in oncology include *CYP2D6*‐5HT3A anti‐emetics/antidepressants (nerve pain)/haloperidol, *CYP2C19*‐proton pump inhibitors/voriconazole/antidepressants, *G6PD*‐rasburicase, and *CYP2D6*/*OPRM1*/*COMT*‐opioids.^11^ DPWG guidelines additionally include oncology specific recommendations for *UGT1A1‐*irinotecan and supportive care recommendations for *HLA‐B*‐allopurinol.^10^, ^12^ Additionally, PGx testing recommendations can also be found in oncology‐specific guidelines, including the National Comprehensive Cancer Network (NCCN) guidelines.^13^

Although there are established benefits of PGx prescribing in oncology, implementation into routine care has lagged behind research evidence and guideline publications.^10^, ^11^, ^12^, ^13^ Despite oncology medicines having good coverage across PGx guidelines, recommendations on actionable prescribing differ, both between PGx guidelines and within oncology treatment guidelines, making translation into practice challenging for healthcare professionals (HCPs). PGx may play a significant role in preventing adverse medicine events for individuals undergoing cancer treatment, as historically their optimization has been complicated by anticancer medicines with a narrow therapeutic index and the potential to cause drug–drug and drug–gene interactions.^7^ Implementation of routine PGx testing in oncology patients to individualize anticancer medicines based on their genetic makeup could greatly improve patient outcomes.^14^

Given the increasing clinical applications of PGx, particularly in oncology, the perspectives of HCPs and consumers could provide insights into the feasibility of establishing widespread PGx screening in practice. The current extent of education and training in this field is also important to inform emerging PGx education practices. To date, current literature concerning PGx perspectives among oncology HCPs and consumers has been limited to primary research conducted in individual institutions.^15^, ^16^ Our study is the first systematic review evaluating PGx perspectives in oncology HCPs and consumers. In this review, we define PGx testing as genetic testing that assesses genetic variations to help predict drug responsiveness and toxicities. Somatic and germline genetic testings to predict cancer risk or tumor mutation which may have already been routinely implemented in oncology practice were not considered as PGx implementation for this review.

The aims of this systematic review were to: (a) evaluate HCPs' and consumers' knowledge, attitudes, and perspectives of PGx screening within the field of oncology; (b) identify the availability and effectiveness of oncology PGx HCP education in improving HCPs' knowledge; and (c) identify the barriers and facilitators to oncology PGx implementation interest and participation.

## METHODS

### Data sources and search strategy

Advanced literature searches were conducted in July 2022 according to Preferred Reporting Items for Systematic reviews and Meta‐Analyses (PRISMA) guidelines.^17^ Articles were identified from the following electronic databases: EMBASE, EMCARE, MEDLINE, and PsycInfo from January 2012 to June 2022. A robust search strategy was devised in consultation with a medical librarian (author S.L.) and adapted to each database with the following keywords included (“pharmacogenetics and pharmacogenomics,” “cancer, tumo(u)r, malignancy, and oncology,” “clinical practice,” “physician, clinician, or clinical staff,” “healthcare professional, doctor, nurse, or pharmacist,” “consumer or patient,” “experience, knowledge, attitudes, or practices,” and “education, training, or learning”). Complete search strategies are provided in Data S1.

Screening was performed by a team of independent reviewers (authors A.B., Z.Y.C., A.H., and J.W.) and articles were vetted through Covidence (Veritas Health Innovation) following strict inclusion and exclusion criteria detailed in the following section. All duplicate articles were removed at this stage. Conflicts that arose at the title, abstract, and full text screening stages were resolved by a third reviewer (authors M.A. or C.X.L.). The review was registered with the International Prospective Register of Systematic Reviews (PROSPERO), registration number CRD42022352348.

### Inclusion and exclusion criteria

Research articles were considered eligible for inclusion if they met the following criteria that were assessed in a hierarchical order: (1) peer‐reviewed primary literature; (2) published between January 2012 and July 2022; (3) written in English; (4) involved individuals diagnosed with cancer (≥18 years old), and HCPs including clinicians, pharmacists, nurses, genetic counselors; and (5) described an aspect of knowledge, attitudes, perspectives, or education regarding PGx in oncology‐based cohorts. Non‐English and out‐of‐date‐range articles were not considered for this review. Articles focused on pediatric cohorts (<18 years old) were excluded due to anticipated differences in treatment settings and needs. Articles were also excluded if they were the incorrect publication type or the full text was not available to the researchers.

### Data extraction strategy

Data were extracted from the eligible articles by a team of independent researchers (authors A.B., Z.Y.C., A.H., and J.W.) with article information added to a common spreadsheet. Data extraction was piloted using a small number of articles and then refined for greater extraction capability after discussions with M.A. and S.H. The following data from selected studies were extracted; study aim, country of study, study design, number of participants, participant characteristics, cancer types and stages, details of PGx testing, and study outcomes. All extracted data were independently reviewed by author C.X.L. then discussed with authors M.A. and S.H.

### Risk of bias assessment and content analysis

The Mixed Methods Appraisal Tool (MMAT) version 2018^18^ was used to assess the quality of the qualitative, quantitative, and mixed methods studies. Three sets of MMAT criteria were used to complete quality assessment: qualitative set of criteria, quantitative descriptive set of criteria, and mixed methods set. Quality assessment of studies was completed by a researcher (authors A.B., Z.Y.C., A.H., or J.W.) and reviewed by author C.X.L. Content analysis was carried out by C.X.L. to identify and code themes and sub‐themes.

## RESULTS

### Study selection

**FIGURE 1:**
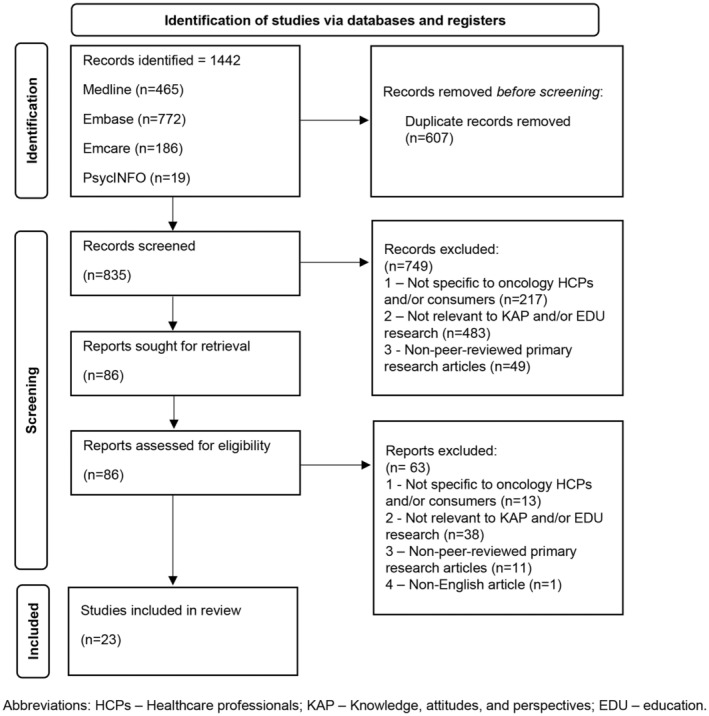
Preferred Reporting Items for Systematic Reviews and Meta‐Analyses (PRISMA) flow diagram.

The predefined search strategy yielded a total of 1442 publications: Medline (*n* = 465), Embase (*n* = 772), EMCARE (*n* = 186), and PsycINFO (*n* = 19; Figure 1). After removing duplicates, 835 publications underwent a two‐step screening and selection process to be assessed for eligibility based on the predetermined inclusion criteria. After excluding publications from title and abstract screening (*n* = 749), those remaining (*n* = 86) underwent full text screening. The final number of publications included in this systematic review was 23,^19^, ^20^, ^21^, ^22^, ^23^, ^24^, ^25^, ^26^, ^27^, ^28^, ^29^, ^30^, ^31^, ^32^, ^33^, ^34^, ^35^, ^36^, ^37^, ^38^, ^39^, ^40^, ^41^ as shown in Figure 1. The majority of the studies met 80% or above of the criteria outlined in the MMAT,^18^ indicating that the articles included in this review were of high quality (Table 1). Ratings across the MMAT domains are detailed in Data File S1.

**TABLE 1:** Characteristics of selected studies.

Studies	Country of study	Study design	Number of participants	Profession(s)	Professional specialty	Consumer group(s)	Cancer type and stage	Details of PGx	Study quality^a^
Professionals only (*n* = 12)									
Devine et al. (2014)^22^	USA	Mixed methods; simulation, survey	10	Doctor	Oncology and other	–	Mixed	*DPYD*‐capecitabine; *UGT1A1*‐irinotecan, nilotinib; *CYP2D6*‐tamoxifen, *TMPT* and *NUDT1*‐6‐mercaptopurine, 6‐thioguanine	80%
Dodson (2014)^23^	USA	Quantitative; survey	368	Nurse	Oncology only	–	Mixed	Nonspecific	80%
Dodson (2015)^24^	USA	Qualitative; survey	246	Nurse	Oncology only	–	Mixed	Nonspecific	100%
Dodson 2018^25^	USA	Quantitative; questionnaire	78	Nurse	Oncology only	–	Mixed	Nonspecific	80%
Dressler et al. (2014)^26^	USA	Quantitative; survey	94	Doctor	Oncology only	–	Mixed	*HER2*, *CYP2D6*, OncotypeDx‐breast cancer; KRAS, OncotypeDx‐colorectal cancer	80%
Jankovic et al. (2019)^30^	18 countries	Quantitative; survey	36	Doctor	Oncology only	–	Lung	Lung‐specific, including *ALK*, *BRAF*, *EGFR*, *HER2*, *KRAS*, *MET*, *ROS1*	40%
Lau‐Min et al. (2022)^31^	USA	Qualitative; interview	25	Doctor, pharmacist	Oncology only	–	Gastrointestinal	*DPYD*‐fluoropyrimidines; *UGT1A1*‐irinotecan	100%
Nagy et al. (2020)^35^	Egypt	Quantitative; survey	184	Doctor, pharmacist	Oncology and other	–	Mixed	Nonspecific	100%
Peppercorn et al. (2013)^37^	USA	Quantitative; survey	201	Doctor	Oncology only	–	Breast	*CYP2D6*‐tamoxifen	80%
Przybylski et al. (2020)^38^	USA	Quantitative; survey	84	Pharmacist	Oncology only	–	Mixed	Nonspecific	80%
Reis et al. (2019)^39^	Portugal	Qualitative; interview	9	Department director, laboratory director	Oncology only	–	Breast	*CYP2D6*–tamoxifen	60%
Zakinova et al. (2019)^41^	USA	Quantitative; survey	135	Doctor, pharmacist	Oncology and other	–	Mixed	42 drug–gene pairs including *CYP2D6*‐tamoxifen; *TPMT*‐mercaptopurine/azathioprine; *DPYD*‐fluoropyrimidines; *CYP3A5*‐tacrolimus; *UGT1A1*‐irinotecan	80%
Consumers only (*n* = 8)									
Ballinger et al. (2017)^19^	USA	Quantitative; survey	417	–	–	Oncology patient	Breast‐Early	rs3125923‐Taxanes; rs28714259‐Anthracyclines	80%
Brewer et al. (2014)^20^	USA	Quantitative; survey	320	–	–	Oncology patient	Breast	*CYP2D6*‐tamoxifen	80%
Cuffe et al. 2014^21^	Canada	Quantitative; survey	278	–	–	Oncology patient	Mixed	Nonspecific	100%
Gray et al. (2012)^27^	USA	Qualitative; interview	69	–	–	Oncology patient	Mixed	Nonspecific	100%
Issa et al. (2013)^29^	USA	Quantitative; questionnaire	300	–	–	Oncology patient	Mixed	*UGT1A1*, *KRAS*‐colorectal cancer; OncotypeDX‐breast cancer	80%
Martin et al. (2016)^33^	Canada	Quantitative; questionnaire	457	–	–	Oncology patient	Mixed	Nonspecific	60%
Meagher et al. (2014)^34^	USA	Qualitative; interview	54	–	–	General public, oncology patient	Mixed	*DPYD*‐fluoropyrimidines	100%
Najafzadeh et al. (2013)^36^	Canada	Quantitative; questionnaire	1096	–	–	General public, oncology patient	Mixed	Nonspecific	100%
Professionals and consumers (*n* = 3)									
Gray et al. (2016)^28^	USA	Mixed methods; survey, interview	194	Doctor	Oncology only	Oncology patient	Mixed‐advanced	Nonspecific	100%
Martens et al. (2019)^32^	Netherlands	Mixed methods; patient records, interviews	753	Doctor, pharmacist, laboratory specialist	Oncology and other	Oncology patient	Mixed	*DPYD*‐fluoropyrimidines	100%
Wu et al. (2017)^40^	USA	Qualitative; interview	26	Doctor, nurse practitioner	Oncology only	Oncology patient	Mixed	*KRAS*‐cetuximab, panitumumab; *HER2*‐trastuzumab, pertuzumab, ado‐trastuzumab emtansine, lapatinib; *BRAF*‐trametinib, dabrafenib, afatinib, erlotinib, panitumumab; *ALK*‐crizotinib; Phildadelphia chromosome‐dasatinib, imatinib, bosutinib, nilotinib; c‐Kit protein, *PDGFR*, and *CD20*‐imatinib, toxitumomab	100%

Abbreviation: PGx, pharmacogenomics.

^a^ Overall study quality scores are presented using the following scale: 100% – All quality criteria met; 80% – 80% quality criteria met; 60% – 60% quality criteria met; 40% – 40% quality criteria met; 20% – 20% quality criteria met.

### Study characteristics

Table 1 shows the characteristics of the 23 studies included for analysis. Of the 23 studies,^19^, ^20^, ^21^, ^22^, ^23^, ^24^, ^25^, ^26^, ^27^, ^28^, ^29^, ^30^, ^31^, ^32^, ^33^, ^34^, ^35^, ^36^, ^37^, ^38^, ^39^, ^40^, ^41^ 12 studies reported findings from HCPs, eight studies focused on consumers only, and the remaining three studies included both HCPs and consumers. The most common methodology was quantitative (*n* = 14), followed by qualitative (*n* = 6) and mixed methods (*n* = 3). Most studies were conducted with HCPs and/or consumers from the United States (*n* = 16). The remainder were conducted in Canada (*n* = 3), Egypt (*n* = 1), Portugal (*n* = 1), the Netherlands (*n* = 1), and one study was conducted with HCPs from 18 countries as part of a multinational consortium‐based project.^30^

### Participant characteristics

Health professionals involved in the selected studies included doctors (*n* = 11), pharmacists (*n* = 5), nurses (*n* = 4), and laboratory director/specialists (*n* = 2; Table 1). Only four out of the 15 studies included HCPs from both oncology and non‐oncology specialties,^22^, ^32^, ^35^, ^41^ with the remaining 11 studies conducted with HCPs involved in cancer care. Two studies – one by Najafzadeh et al.^36^ and the other by Meagher et al.^34^ – recruited participants from the general public with the former recruiting the highest number of participants (*n* = 1096) across all 23 studies.^36^ The qualitative study by Reis et al.^39^ had the lowest number of participants (*n* = 9).

### Types of cancer and PGx testing

Most of the studies were not specific to a single tumor stream (*n* = 17). Of the remaining studies, four focused on breast cancer, and one each on lung and gastrointestinal cancers (Table 1). Only two studies specified the stages of cancer investigated.^19^, ^28^ A range of PGx testing was included in the selected studies, however, 10 studies did not investigate or provide details of PGx testing, instead investigating the broader concept of PGx. The most common tests included *CYP2D6* for tamoxifen (*n* = 6), *DPYD* for fluoropyrimidines (*n* = 5), and *UGT1A1* for irinotecan (*n* = 4). Zakinova et al.^41^ investigated 42 drug–gene pairs in their survey questions. Two studies, Dressler et al.^26^ and Issa et al.,^29^ included genomic biomarker prognostic assays for breast and colorectal cancer, OncotypeDx, along with other PGx tests in their surveys.

### Summary of study findings

Twenty‐two studies reported knowledge, attitudes, and perspectives of oncology HCPs and/or consumers toward PGx (Table 2). The remaining study by Dodson designed and evaluated a purpose‐built PGx education module that significantly improved PGx knowledge in nurses (*p* < 0.01).^25^ The mean pre‐module score was 72.7 out of 100 in this study,^25^ indicating good baseline knowledge about genomics and PGx testing in this cohort. Overall, both HCPs and consumers perceived PGx in oncology positively, in particular the potential of PGx in guiding cancer treatment to improve efficacy and minimize toxicity.^19^, ^20^, ^21^, ^22^, ^23^, ^24^, ^26^, ^27^, ^28^, ^29^, ^30^, ^32^, ^35^, ^37^, ^39^, ^40^, ^41^ It was found that there is a high level of acceptance of PGx testing (~90% across studies) and information among consumers, including those who had PGx testing and those who had not had PGx testing and were asked hypothetical questions about PGx,^19^, ^20^, ^21^, ^27^, ^28^, ^29^, ^32^, ^40^ and two studies reported that patients with cancer expressed high willingness to pay for PGx tests.^21^, ^29^

**TABLE 2:** Summary of studies.

Studies	Aim	Study theme(s)	Study findings
Professionals only (*n* = 12)			
Devine et al. (2014)^22^	Useability of PGx alerts and impact on prescribing choice	KAP	**Facilitators** Succinct, simple, and relevant dosing guidelines Education on interpretation and clinical applicability Access to credible resources
Dodson (2014)^23^	Awareness, perspectives and support of PGx	KAP and EDU	**Outcomes (quantitative data)** 25% perceived poor knowledge 38% had prior PGx education 89% saw the need for PGx **Summary** Overall positive attitude toward PGx testing in oncology Prior genomic and PGx education, oncology certification and prior experience led to higher perceived and actual PGx knowledge
Dodson (2015)^24^	Concerns and opinions of PGx	KAP	**Facilitators** Consistency of PGx testing Utility of PGx testing Availability of genetic counseling Education **Barriers** Costs Health inequity for uninsured patients
Dodson (2018)^25^	Efficacy of PGx education for oncology nurses	EDU	**Outcomes (quantitative data)** Mean pre‐module score: 72.7/100 Mean post‐module score: 85.9/100; *p* < 0.01 (*n* = 78) **Summary** Continuing PGx education module significantly improved PGx knowledge
Dressler et al. (2014)^26^	Attitudes of oncologists toward adoption of cancer PGx	KAP and EDU	**Outcomes (quantitative data)** 98% agreed with the potential of PGx in guiding treatment 24% were comfortable with their knowledge and interpretation skills 94% desired cancer PGx education **Facilitators** Continuing education Experience Coordinated infrastructure to disseminate and facilitate use of accurate PGx information **Barriers** Non‐standardized nomenclature Lack of preparedness
Jankovic et al. (2019)^30^	Research and practices related to lung cancer diagnosis/therapy on global scale	KAP	**Outcomes (quantitative data)** >90% were guided by PGx results in targeted therapy prescribing 96% wanted more biomarkers included in routine PGx testing
Lau‐Min et al. (2022)^31^	Attitudes and barriers to PGx implementation	KAP	**Barriers** Perceived lack of evidence^a^ Uncertain impact on clinical care Inefficient testing process Lack of alternative therapeutic options Costs Knowledge and interpretation of tests Self‐efficacy of testing ordering
Nagy et al. (2020)^35^	Knowledge, attitudes, and perspectives of pharmacists and physicians on PGx testing	KAP and EDU	**Outcomes (quantitative data)** Low PGx knowledge scores among Egyptian pharmacists and physicians No difference in knowledge across different professions, specialties, experience and education levels (*p* > 0.05) Pharmacists felt more responsible in implementing PGx‐guided treatment (*p* < 0.001), more favorable to include PGx data in patient databases (*p* < 0.0001), had a higher interest in PGx education and training than physicians (*p* < 0.0001) **Barriers** Lack of knowledge/skill Lack of infrastructure Funding, shortage of qualified staff Lack of clinical guidelines^a^ Time Cultural or religious beliefs Patient refusal
Peppercorn et al. (2013)^37^	Knowledge, practice, and attitudes toward PGx (*CYP2D6*) testing	KAP	**Outcomes (quantitative data)** Majority (56%) were willing to order PGx test if requested by a patient and likely to change treatment based on test results **Facilitators** Professional guidelines^b^ Randomized controlled trial evidence Clear clinical utility of test results Insurance coverage Authority approved tests
Przybylski et al. (2020)^38^	Knowledge, challenges, and roles of pharmacists in PGx practices	KAP and EDU	**Outcomes (quantitative data)** Pharmacists were comfortable with assessing PGx data, which were positively associated with experience (*p* = 0.02), post‐graduate training (*p* = 0.04), tumor board participation (*p* = 0.01) and involvement in internal PGx policies development (*p* = 0.02) PGx education most commonly received through work (64%), continuing education (64%) and pharmacy school (42%) **Barriers** Lack of clinical decision support tools Poor PGx results visibility in health records Insurance denials Long processing time
Reis et al. (2019)^39^	Knowledge, practice and attitudes toward PGx testing	KAP	**Barriers** Cost Unclear clinical benefits Lack of unequivocal clinical decision‐making guidelines **Summary** All stakeholders agreed PGx is key to predicting potential drug reactions
Zakinova et al. (2019)^41^	Practices, needs, and interests to PGx	KAP	**Outcomes (quantitative data)** One‐third of physicians (29%) and pharmacists (32%) currently use PGx in practice Majority of physicians (63%) and pharmacists (54%) expressed interest in integrating PGx 59% of both professions were comfortable with evidence obtained through observational studies **Barriers** Lack of established and clear clinical guidelines^a^ Limited continuing education Cost
Consumers only (*n* = 8)			
Ballinger et al. (2017)^19^	PGx impact on patient Tx choice	KAP	**Summary** Biomarker risk profiles for chemotherapy toxicity changed patients' treatment choices
Brewer et al. (2014)^20^	Perceived impact of PGx on Tx outcomes	KAP	**Outcomes (quantitative data)** *CYP2D6* genotype altered patients' perception of recurrence risk when prescribed tamoxifen **Summary** High receptiveness to genomic risk information
Cuffe et al. (2014)^21^	Knowledge, attitudes, and preferences for PGx testing	KAP	**Outcomes (quantitative data)** Majority of patients were accepting of PGx testing (acceptance rate: 97%–99%) Out of pocket costs (CAD 1000–2000) and delays in treatment to avail of testing (14–16 days) 22% lack understanding of PGx testing **Summary** Shared decision making on PGx testing was highly desired
Gray et al. (2012)^27^	Attitudes of patients toward somatic PGx	KAP	**Barriers** Concerns over incidental information Information overload, unclear testing benefits Cost Discrimination **Summary** High willingness for PGx testing, with most participants reported willingness to undergo somatic or germline PGx testing
Issa et al. (2013)^29^	Patient preferences for OncotypeDX in breast and colorectal cancer	KAP	**Outcomes (quantitative data)** Breast cancer patients were more concerned about undertreatment due to a false‐negative result than overtreatment due to a false‐positive result (*p* = 0.0002), the opposite was observed in colorectal cancer patients (*p* = 0.0024) **Summary** Patients had a high willingness to pay and regarded test accuracy as an important attribute in decision‐making Colorectal cancer patients less knowledgeable about PGx than breast cancer patients
Martin et al. (2016)^33^	Impact of survey format preference on PGx testing understanding	KAP	**Summary** Survey format (computerized and print) did not impact patient understanding of PGx testing
Meagher et al. (2014)^34^	Biobank participants' understanding of PGx	KAP	**Summary** Participants generally understand their PGx results PGx disclosure in biobanking can involve participants conflation of drug‐gene interactions as allergies or other medical conditions A functional framework of PGx recipient communication to experts could support bidirectional communication between experts and PGx recipients, increase PGx recipients' familiarity with biomedical vocabulary to improve genetic literacy, and clarify how PGx results will be recorded in the patients' medical record
Najafzadeh et al. (2013)^36^	Preferences of PGx testing in relation to cancer prognosis	KAP	**Outcomes (quantitative data)** Patients were more concerned of test sensitivity (*p* < 0.001) and preferred opting‐out of PGx testing than public (*p* = 0.0002) when test sensitivity is lacking **Summary** Preferences for PGx‐guided treatment were influenced by test sensitivity, type, and prognosis of cancer
Professionals and consumers (*n* = 3)			
Gray et al. (2016)^28^	Knowledge, attitudes, and perceptions of oncologists and patients toward whole‐exome sequencing PGx testing	KAP and EDU	**Outcomes (quantitative data)** High confidence levels among oncologists for PGx implementation and 78% supported whole‐exome sequencing finding disclosure if clinical validity is established Patients had moderately low PGx knowledge, most (84%–99%) wanted most cancer‐related and incidental WES results **Barriers** Data interpretation Disclosing non‐cancer findings Determining the ‘actionability’ of alterations Lack of education Managing patient and family expectations and emotional response
Martens et al. (2019)^32^	DPD testing in relation to current pharmacogenetic practice	KAP	**Outcomes (quantitative data)** Update of national guideline for colorectal cancer led to 86% increase in DPD testing from Q2‐2017 (1/86) to Q4‐2018 (73/84) **Facilitators** Clear evidence Professional guidelines^c^ Peer influence Streamlined procedures for test ordering and reporting Education Information provided in simple language for patients **Barriers** Lack of clear division of responsibilities Long processing time Complex IT infrastructures
Wu et al. (2017)^40^	Perspectives of guideline recommended PGx tests	KAP	**Facilitators** Clinical benefits Medicine subsidies Availability/access to testing Streamlined authorisation procedures **Barriers** Long processing time Complex ordering system Insurance coverage Costs for gene panel tests **Summary** High acceptability of PGx testing in both providers and patients

Abbreviations: CAD, Canadian dollar; DPD, dihydropyrimidine dehydrogenase; EDU, education; IT, information technology; KAP, knowledge, attitudes, perspectives; PGx, pharmacogenomics; Tx, treatment; WES, whole exome sequencing.

^a^ PGx guidelines existed at the time of the studies however participants were unaware or unconvinced.

^b^ PGx guidelines were not available at the time of the study and hence call for guidelines by participants.

^c^ The impact of PGx guideline implementation on PGx testing uptake was assessed.

Two studies, Dodson and Przybylski et al.,^23^, ^38^ assessed the prior PGx education HCPs had received and found that most had no formalized PGx education during their undergraduate training. Four studies reported low knowledge among HCPs and a high desire for further education in cancer PGx.^23^, ^26^, ^35^, ^38^ Similarly, Gray et al. reported moderately low PGx knowledge (mean score of 4 out of 7) among oncology patients.^28^

Two major themes were derived from the content analysis of all 23 studies, namely barriers and facilitators of PGx in oncology. For each theme, six sub‐themes were identified and are detailed in the following sections.

### Barriers

#### Cost, lack of insurance coverage, and resources

A significant barrier to routine PGx implementation in oncology practice is the cost of testing, which was reported in eight studies.^21^, ^24^, ^27^, ^31^, ^35^, ^38^, ^39^, ^40^, ^41^ Both oncology HCPs and patients voiced their concerns about the cost and lack of health insurance coverage for PGx tests that impede the utilization of PGx screening in routine clinical care, despite high willingness for PGx‐guided oncology treatment. Lack of PGx testing infrastructure to support integration of PGx into clinical oncology practice was also raised as a major barrier.

#### Lack of PGx clinical guidelines and perceived benefits

In four studies,^31^, ^35^, ^39^, ^41^ lack of PGx clinical guidelines were cited by HCPs as one of the hurdles in adopting PGx in oncology practice. HCPs were concerned about the lack of data from randomized controlled clinical trials and conflicting recommendations from national clinical oncology guideline bodies, such as NCCN and European Society for Medical Oncology.^31^, ^39^ Some HCPs perceived that there were no clear benefits of PGx testing given the lack of consistency of recommendations across different guidelines. In a study conducted by Reis et al.,^39^ an HCP stated that, “We don't apply the test because there is no international recommendation for its implementation and the results of several clinical trials are still contradictory and therefore is no consensus on its use.” HCPs were also concerned about the lack of alternative therapeutic options for patients who received their PGx test results, which resulted in diminished perceived values of PGx testing and reduced motivations to promote PGx. Similarly, in an interview study involving oncology patients, participants reported that they were reluctant to take PGx tests if test benefits are unclear.^27^

#### Lack of PGx knowledge, skills, and education

Limited PGx integration into routine oncology practice was attributed to lack of PGx knowledge, skills, and continuing professional education in five studies.^26^, ^28^, ^31^, ^35^, ^41^ The uncertainty of how to interpret test results and enact PGx recommendations into their practice among HCPs were found to be common barriers, with the majority of HCPs having not received PGx education as part of their undergraduate training.

#### Complex PGx test ordering system and long processing time

HCPs appeared to be frustrated with the lack of PGx integration into routine oncology practice, with complex PGx test ordering processes and long processing time identified as hurdles of PGx implementation in five studies.^26^, ^31^, ^32^, ^38^, ^40^ PGx testing and result reporting systems were described as inefficient, having non‐standardized nomenclature, and not fully integrated with electronic medical records, adding to the burden of HCPs to implement PGx in oncology.

#### Health inequity, discrimination, and privacy concern

Health inequity for patients who were uninsured, fear of being discriminated by employers due to PGx testing data, and privacy issues were highlighted in two studies.^24^, ^27^ In a study conducted by Gray et al.,^27^ people with cancer also reported concerns about how incidental data from PGx testing could be handled.

#### Health beliefs and self‐efficacy

Only one study reported that HCPs may be less inclined to adopt PGx testing in their practice due to cultural or religious beliefs, however, these were not described further.^35^ Self‐efficacy of test ordering was identified as one of the barriers in a study conducted by Lau‐Min et al.,^31^ as this practice was not incorporated in their current clinical workflows.

### Facilitators

#### Oncology PGx education

It is evident that PGx education is a key driver to promoting PGx integration in clinical oncology.^22^, ^23^, ^24^, ^25^, ^26^, ^32^, ^38^ Across studies, most HCPs expressed a high desire for cancer PGx education as part of their continuing professional education. Notably, Dodson undertook a comparison of oncology nurses' knowledge score pre‐ and post‐education intervention and demonstrated that continuing PGx education is effective in improving nurses' short‐term knowledge of genomic and PGx information (Table 2).^25^

#### Evidence‐based PGx guidelines with clear clinical impact

Another major facilitator driving the implementation of PGx in oncology is evidence‐based PGx guidelines. Oncology HCPs viewed consensus guidelines with evidence from randomized controlled trials and clear impact on patient outcomes as instrumental in promoting use of PGx testing in routine oncology practice.^22^, ^24^, ^32^, ^37^ Succinct and concise PGx‐guided dosing guidelines embedded in electronic health records was also recommended by HCPs.^22^

#### Streamlined and regulated PGx testing

Streamlined PGx ordering systems and authority‐regulated testing were found to be important to facilitate PGx integration into routine practice.^24^, ^32^, ^37^, ^40^ HCPs regarded consistency of PGx testing, test validity, usability, and integrated authorization procedures as enablers.

#### Funding and access to resources

Access to credible resources and coordinated dissemination of PGx resources through local professional oncology networks were described in two studies as facilitators.^22^, ^26^ Funding for PGx‐guided medicines was identified to be a key aspect in promoting PGx implementation in oncology in a mixed‐cohort study conducted by Wu et al.^40^

#### Patient requests and professional engagement

Patient requests could facilitate increased PGx uptake in clinical oncology practice.^32^ Peppercorn et al.^37^ reported that the majority of clinicians surveyed were willing to order PGx assays if requested by patients. Przybylski et al.^38^ found that engagement with internal PGx policy development and tumor board participation were positively associated with pharmacists' comfort in PGx result interpretation (*p* = 0.01), suggesting that professional PGx engagement opportunities could promote adoption of PGx in oncology practice by HCPs.

#### Effective communication with consumers

In two studies where a mixed cohort of consumers and HCPs were recruited, effective communication was identified as an enabler to increase adoption of oncology‐focused PGx services. Martens et al.^32^ described that simple wording should be used to report PGx results for consumers. Meagher et al.^34^ developed a functional framework to support bidirectional communication between HCPs and consumers, where patients' word choices can be mapped to different communicative functions by HCPs, thus allowing HCPs to anticipate and bridge the terminology disconnect in clinical encounters and facilitate effective communication when discussing PGx results with patients.

## DISCUSSION

This is the first systematic review to explicitly explore HCPs' and consumers' knowledge, attitudes, perspectives, and education needs for PGx in adult oncology care. Previous reviews included studies reporting on PGx across different specialities.^15^, ^42^ Our discipline‐specific approach allows identification for actionable change and advancement of PGx in oncology with greater precision. Furthermore, few studies have evaluated both consumers' and HCPs' perspectives on PGx testing concomitantly.^28^, ^32^, ^40^ Thus, by including both groups of stakeholders and considering their perspectives equally, this review serves to provide realistic perspectives on the current gaps and ways to foster acceptance and meaningful utilization of PGx in standard oncology practice.

Analysis of the included studies identified that current evidence is primarily reflective of American and Canadian populations. Most of the included studies typically included HCPs and consumers from mixed oncology subspecialties. It is possible that the findings may not be generalizable to all HCPs and consumers in oncology as there is evidence that differences exist in the knowledge and practices across different subspecialities, professions, and work settings.^26^, ^29^, ^35^

In line with previous studies in noncancer‐specific settings,^15^, ^43^, ^44^, ^45^ the results of this systematic review reinforce the notion that utility of PGx testing in oncology is perceived positively by both HCPs and patients. Costs and lack of insurance coverage were found to be the main barriers in adoption of PGx testing in oncology. As public health coverage, for example, Medicare (USA) for PGx tests is limited to patients with certain conditions, many patients rely on reimbursements from insurance companies for PGx tests.^46^ In the United States, it has been reported that coverage of clinically relevant PGx tests by insurance companies is becoming more widely available. However, coverage can vary greatly between different insurance providers which can limit access to PGx testing, especially for patients from disadvantaged backgrounds.^46^

Resource constraints, such as lack of trained staff and testing facilities, unfortunately impede the implementation of PGx in Egypt specifically, a lower‐middle income country, which were not reported in the remaining 22 studies conducted in high‐income countries as defined by the World Bank.^47^ It is worth noting that a lack of access to PGx testing is also a known issue in high income countries, as several studies in the United States have reported that rural populations have limited access to PGx testing and PGx‐trained HCPs compared to people residing in metropolitan areas, due to the fact that most PGx services have been limited to larger institutions.^48^, ^49^ In the study conducted by Nagy et al. in Egypt, they reported cultural or religious beliefs as a barrier to adoption of PGx in oncology practice, which was not identified in the other studies included in this review; however, this barrier was not further explained by the authors. These findings highlight that a unique set of challenges exist in different regions, and further studies outside of the United States, particularly in emergent nations and within different health system constructs, are required to better understand patients and practitioners' viewpoints and education needs in oncology PGx relevant to the local context.

It was evident that the key driver of successful PGx implementation in oncology is education. Although only a single study evaluated the effectiveness of a purpose‐built PGx education module in improving HCPs' PGx knowledge, continuing professional PGx education was found to be desired by oncology HCPs in multiple studies.^26^, ^28^, ^35^ Most HCPs reported that they had limited exposure to PGx education during their undergraduate studies and mainly received PGx‐specific education on the job or through post‐graduate training. Similar findings were also reported in a recent Australian study conducted by Pearce et al. involving non‐oncology‐specific HCPs, where only less than a quarter of the HCPs surveyed received PGx training as part of their undergraduate curricula.^43^ This is reasonable given PGx was/is an emerging field despite personalized medicine being recognized for its potential over 2 decades ago. PGx education in the form of continuing professional education to equip oncology HCPs with requisite translational knowledge and skills, holds the key to bridge the gap in current practice.

Other key drivers for HCPs to drive PGx‐integrated practice identified in this review include consistency of information across evidence‐based PGx guidelines with demonstration of impact on patient outcomes and streamlined PGx testing processes. There is potential to promote both drivers through increased collaboration of oncology HCPs, laboratory specialists, and researchers in sharing information and resources to shed light on current inconsistencies between different guidelines and come to agreement on consensus guidelines/a position statement and testing procedures. It must be noted that conflicting evidence and recommendations exist even for the most reported PGx test in this review, *CYP2D6*, whereby dosage adjustments for tamoxifen based on CYP2D6 phenotype is recommended by the CPIC,^11^ whereas NCCN guidelines^13^ recommend against the use of *CYP2D6* testing to determine tamoxifen dosing. This highlights that HCPs cannot simply rely on evidence‐based guidelines to implement PGx in practice. Rather, HCPs must learn how to interpret PGx data and able to communicate effectively with patients around decisions to utilize PGx to inform dosing, as PGx will be increasingly available in clinical care regardless of availability of consistent recommendations across different PGx guidelines.

A strength of this study included the systematic search strategy that followed the PRISMA guidelines for conducting systematic reviews and the robust screening strategy based on a hierarchical order of criteria to minimize inconsistencies in searching and screening of articles. It is worth noting that in Dressler et al.^26^ and Issa et al.,^29^ OncotypeDx genomic biomarker prognostic assays for breast and colorectal cancer were included in their surveys as PGx tests to investigate PGx acceptance and adoption among HCPs and consumers. Both studies were included in this review on the basis that other PGx tests used to guide dosing and selection of medicines, such as *CYP2D6*, *UGT1A1*, *HER2*, and *KRAS*, were described in the studies.

The exclusion of research studies involving non‐oncology HCPs and consumers may be considered as a limitation, as non‐oncology‐specific studies, such as the Australian study by Pearce et al.,^43^ may contribute to local learning on integrating PGx into routine cancer care. Another limitation of this systematic review is that some of the selected studies were published 8–10 years ago, and findings from these studies may not reflect the current practice of PGx in oncology as it is informed by a fast‐moving field of research. Nonetheless, given that most countries have just started to integrate PGx testing in their health systems, insights from older studies in countries that are leading PGx research and practice will be relevant for other countries to implement oncology‐focused PGx services in the near future.

This review was limited to oncology because oncology clinical care pathways differ from other acute/chronic disease settings. By focusing on this disease setting, we provide contextually relevant findings, but acknowledge there may be cross‐over in themes and opportunities to learn from experiences in other disease groups.

## CONCLUSION

Findings from this review shed light on the main barriers in global oncology PGx implementation, and facilitators that are key in mapping ways to inform implementation strategies that would increase PGx uptake in oncology practice. It is apparent that consensus PGx guidelines relevant to local context and PGx education in oncology are salient in bridging the current gaps in practice. Future work aiming at providing continuing professional PGx education is warranted to improve oncology HCPs' knowledge and skills in PGx. Development of consistent recommendations across PGx clinical guidelines and streamlined PGx ordering systems for local institutions, drawing on high quality evidence from randomized controlled trials, will be important in promoting clinical implementation of PGx by oncology HCPs and uptake by consumers.

## AUTHOR CONTRIBUTIONS

C.X.L., A.B., Z.Y.C., A.H., J.W., S.L., S.Y.H., and M.A. wrote the manuscript. C.X.L., S.L., S.Y.H., and M.A. designed the research. C.X.L., A.B., Z.Y.C., A.H., J.W., S.L., and M.A. performed the research. C.X.L., A.B., Z.Y.C., A.H., J.W., S.Y.H., and M.A. analyzed the data.

## FUNDING INFORMATION

This research was undertaken under a program funded by the Australian Medical Research Future Fund Quality, Safety, and Effectiveness of Medicine Use by Pharmacists (grant ID: MRFMMIP000011, CIA M Alexander).

## CONFLICT OF INTEREST STATEMENT

The authors declared no competing interests for this work.

## Supporting information


Data S1

Data S2

Table S1
