# Supplementary material for: Healthcare professionals' and consumers' knowledge, attitudes, perspectives, and education needs in oncology pharmacogenomics: A systematic review
Source: Clin Transl Sci. 2023 Nov 22;16(12):2467–82. doi: 10.1111/cts.13672 (PMC10719462; doi:10.1111/cts.13672)
Supplement: Supplementary file 1 — Data S1 [file CTS-16-2467-s001.pdf]

Database: Embase <1974 to 2022 July 15>
Search Strategy:
----
1 exp pharmacogenomics/ or exp pharmacogenetic testing/ or exp pharmacogenetics/ (34701)
2 (pharmacogenetic* or pharmacogenomic* or pgx or capgx).tw,kf. (30822)
3 1 or 2 (45091)
4 exp neoplasms/ (5126116)
5 (cancer* or carcinoma* or neoplas* or tumo?r* or malignan* or oncolog* or metasta* or leuk?emia* or lymphoma*).tw,kf. (5413056)
6 4 or 5 (6406862)
7 exp clinical practice/ or exp attitude to health/ or exp health personnel attitude/ (621665)
8 ((physician* or clinician* or clinical staff or clinical practice or pharmacist* or pharmacy or pharmacies or oncologist* or oncology provider* or doctor* or nurse* or health professional* or health care professional* or healthcare professional* or healthcare worker* or health care worker* or healthcare provider* or health care provider* or healthcare personnel or health care personnel or health personnel or medical staff or medical practitioner* or general practitioner* or genetic counsellor* or care-giver* or caregiver* or carer* or patient* or consumer* or participant*) adj7 (knowledge* or understanding or misunderstanding or comprehension or comprehend* or experience or experiences or expertise or receptive* or perspective* or perception* or attitude* or opinion* or accepta* or utili#ation or utility or utili#e or utili#ing or preference* or practice* or education or training or learning or barrier* or difficult* or aware* or engagement or readiness or interest or interests or willingness or unwillingness or implement* or endorse* or support* or participation or embrace* or familiari* or enthusias* or motivat* or confidence)).tw,kf. (1952022)
9 7 or 8 (2300001)
10 3 and 6 and 9 (1686)
11 (exp child/ or exp pediatrics/ or exp infant/ or exp adolescent/) not exp adult/ (2400676)
12 ((child* or infant* or newborn* or p?ediatric* or juvenile* or adolescen* or teen* or youth) not adult*).ti. (1644390)
13 conference abstract.pt. (4451987)
14 11 or 12 or 13 (6875075)
15 10 not 14 (1289)
16 limit 15 to (english language and yr="2012 -Current") (772)

****

Database: Ovid Emcare <1995 to 2022 Week 28>
Search Strategy:
----
1 exp pharmacogenomics/ or exp pharmacogenetic testing/ or exp pharmacogenetics/ (5460)
2 (pharmacogenetic* or pharmacogenomic* or pgx or capgx).tw,kf. (4430)
3 1 or 2 (6981)
4 exp neoplasms/ (741075)
5 (cancer* or carcinoma* or neoplas* or tumo?r* or malignan* or oncolog* or metasta* or leuk?emia* or lymphoma*).tw,kf. (822487)
6 4 or 5 (982463)
7 exp clinical practice/ or exp attitude to health/ or exp health personnel attitude/ (200853)
8 ((physician* or clinician* or clinical staff or clinical practice or pharmacist* or pharmacy or pharmacies or oncologist* or oncology provider* or doctor* or nurse* or health professional* or health care professional* or healthcare professional* or healthcare worker* or health care worker* or healthcare provider* or health care provider* or healthcare personnel or health care personnel or health personnel or medical staff or medical practitioner* or general practitioner* or genetic counsellor* or care-giver* or caregiver* or carer* or patient* or consumer* or participant*) adj7 (knowledge* or understanding or misunderstanding or comprehension or comprehend* or experience or experiences or expertise or receptive* or perspective* or perception* or attitude* or opinion* or accepta* or utili#ation or utility or utili#e or utili#ing or preference* or practice* or education or training or learning or barrier* or difficult* or aware* or engagement or readiness or interest or interests or willingness or unwillingness or implement* or endorse* or support* or participation or embrace* or familiari* or enthusias* or motivat* or confidence)).tw,kf. (700579)
9 7 or 8 (806574)
10 3 and 6 and 9 (295)
11 (exp child/ or exp pediatrics/ or exp infant/ or exp adolescent/) not exp adult/ (617515)
12 ((child* or infant* or newborn* or p?ediatric* or juvenile* or adolescen* or teen* or youth) not adult*).ti. (540758)
13 11 or 12 (799697)
14 10 not 13 (277)
15 limit 14 to (english language and yr="2012 -Current") (186)

****

Database: Ovid MEDLINE(R) ALL <1946 to July 15, 2022>
Search Strategy:
----
1 exp pharmacogenetics/ or exp pharmacogenomic testing/ (13897)
2 (pharmacogenetic* or pharmacogenomic* or pgx or capgx).tw,kf. (19870)
3 1 or 2 (24696)
4 exp neoplasms/ (3711765)
5 (cancer* or carcinoma* or neoplas* or tumo?r* or malignan* or oncolog* or metasta* or leuk?emia* or lymphoma*).tw,kf. (4127165)
6 4 or 5 (4938577)
7 exp Practice Patterns, Physicians'/ or exp Health Knowledge, Attitudes, Practice/ or exp "Attitude of Health Personnel"/ (332607)
8 ((physician* or clinician* or clinical staff or clinical practice or pharmacist* or pharmacy or pharmacies or oncologist* or oncology provider* or doctor* or nurse* or health professional* or health care professional* or healthcare professional* or healthcare worker* or health care worker* or healthcare provider* or health care provider* or healthcare personnel or health care personnel or health personnel or medical staff or medical practitioner* or general practitioner* or genetic counsellor* or care-giver* or caregiver* or carer* or patient* or consumer* or participant*) adj7 (knowledge* or understanding or misunderstanding or comprehension or comprehend* or experience or experiences or expertise or receptive* or perspective* or perception* or attitude* or opinion* or accepta* or utili#ation or utility or utili#e or utili#ing or preference* or practice* or education or training or learning or barrier* or difficult* or aware* or engagement or readiness or interest or interests or willingness or unwillingness or implement* or endorse* or support* or participation or embrace* or familiari* or enthusias* or motivat* or confidence)).tw,kf. (1327137)
9 7 or 8 (1540695)
10 3 and 6 and 9 (755)
11 (exp child/ or exp pediatrics/ or exp infant/ or exp adolescent/) not exp adult/ (2084178)
12 ((child* or infant* or newborn* or p?ediatric* or juvenile* or adolescen* or teen* or youth) not adult*).ti. (1420028)
13 11 or 12 (2460382)
14 10 not 13 (710)
15 limit 14 to (english language and yr="2012 -Current") (465)

****

Database: APA PsycInfo <1806 to July Week 2 2022>
Search Strategy:
----
1 (pharmacogenetic* or pharmacogenomic* or pgx or capgx).mp. (2341)
2 exp neoplasms/ (57630)
3 (cancer* or carcinoma* or neoplas* or tumo?r* or malignan* or oncolog* or metasta* or leuk?emia* or lymphoma*).mp. (102417)
4 2 or 3 (102624)
5 exp clinical practice/ or exp Health Personnel Attitudes/ or exp Health Attitudes/ or exp Client Attitudes/ or exp Health Knowledge/ (87488)
6 ((physician* or clinician* or clinical staff or clinical practice or pharmacist* or pharmacy or pharmacies or oncologist* or oncology provider* or doctor* or nurse* or health professional* or health care professional* or healthcare professional* or healthcare worker* or health care worker* or healthcare provider* or health care provider* or healthcare personnel or health care personnel or health personnel or medical staff or medical practitioner* or general practitioner* or genetic counsellor* or care-giver* or caregiver* or carer* or patient* or consumer* or participant*) adj7 (knowledge* or understanding or misunderstanding or comprehension or comprehend* or experience or experiences or expertise or receptive* or perspective* or perception* or attitude* or opinion* or accepta* or utili#ation or utility or utili#e or utili#ing or preference* or practice* or education or training or learning or barrier* or difficult* or aware* or engagement or readiness or interest or interests or willingness or unwillingness or implement* or endorse* or support* or participation or embrace* or familiari* or enthusias* or motivat* or confidence)).mp. (502141)
7 5 or 6 (526633)
8 1 and 4 and 7 (34)
9 ((child* or infant* or newborn* or p?ediatric* or juvenile* or adolescen* or teen* or youth) not adult*).ti. (547320)
10 8 not 9 (33)
11 limit 10 to (english language and yr="2012 -Current") (19)

****
