# Supplementary material for: Healthcare professionals' and consumers' knowledge, attitudes, perspectives, and education needs in oncology pharmacogenomics: A systematic review
Source: Clin Transl Sci. 2023 Nov 22;16(12):2467–82. doi: 10.1111/cts.13672 (PMC10719462; doi:10.1111/cts.13672)
Supplement: Supplementary file 3 — Table S1 [file CTS-16-2467-s003.docx]

**Supplementary Data 2**

Table S1: Risk of bias assessment for qualitative studies using MMAT

| Studies | Is the qualitative approach appropriate to answer the research question? | Are the qualitative data collection methods adequate to address the research question? | Are the findings adequately derived from the data? | Is the interpretation of results sufficiently substantiated by data? | Is there coherence between qualitative data sources, collection, analysis and interpretation? | Percentage of criteria met (%) |
| --- | --- | --- | --- | --- | --- | --- |
| Dodson 2015 | Yes | Yes | Yes | Yes | Yes | 100 |
| Gray et.*al.* 2012 | Yes | Yes | Yes | Yes | Yes | 100 |
| Lau-Min et.*al.* 2022 | Yes | Yes | Yes | Yes | Yes | 100 |
| Meagher et.*al.* 2022 | Yes | Yes | Yes | Yes | Yes | 100 |
| Reis et.*al.* 2019 | Yes | Yes | No | No | Yes | 60 |
| Wu et.*al.* 2017 | Yes | Yes | Yes | Yes | Yes | 100 |

Table S2: Risk of bias assessment for quantitative studies using MMAT

| Studies | Is the sampling strategy relevant to address the research question? | Is the sample representative of the target population? | Are the measurements appropriate? | Is the risk of nonresponse bias low? | Is the statistical analysis appropriate to answer the research question? | Percentage of criteria met (%) |
| --- | --- | --- | --- | --- | --- | --- |
| Ballinger et.*al.* 2017 | Yes | Yes | Yes | Can’t tell | Yes | 80 |
| Brewer et.*al.* 2014 | Yes | Yes | Can’t tell | Yes | Yes | 80 |
| Cuffe et.*al.* 2014 | Yes | Yes | Yes | Yes | Yes | 100 |
| Dodson 2014 | Yes | Yes | Yes | No | Yes | 80 |
| Dodson 2018 | Yes | Yes | Yes | No | Yes | 80 |
| Dressler et.*al.* 2014 | Yes | Yes | Yes | No | Yes | 80 |
| Issa et.*al.* 2013 | Yes | Yes | Yes | No | Yes | 80 |
| Jankovic et.*al.* 2019 | Yes | Yes | Can’t tell | Can’t tell | Can’t tell | 40 |
| Martin et.*al.* 2016 | Yes | Yes | Can’t tell | Can’t tell | Yes | 60 |
| Nagy et.*al.* 2020 | Yes | Yes | Yes | Yes | Yes | 100 |
| Najafzadeh et.*al.* 2013 | Yes | Yes | Yes | Yes | Yes | 100 |
| Peppercorn et.*al.* 2013 | Yes | Yes | Yes | No | Yes | 80 |
| Przybylski et.*al.* 2020 | Yes | Yes | Yes | No | Yes | 80 |
| Zakinova et.*al.* 2019 | Yes | Yes | Yes | No | Yes | 80 |

Table S3: Risk of bias assessment for mixed methods studies using MMAT

| Assessment questions | Devine et.*al.* 2014 | Gray et.*al.* 2016 | Martens et.*al.* 2019 |
| --- | --- | --- | --- |
| Is the qualitative approach appropriate to answer the research question? | Yes | Yes | Yes |
| Are the qualitative data collection methods adequate to address the research question? | Yes | Yes | Yes |
| Are the findings adequately derived from the data? | Yes | Yes | Yes |
| Is the interpretation of results sufficiently substantiated by data? | Yes | Yes | Yes |
| Is there coherence between qualitative data sources, collection, analysis and interpretation? | Yes | Yes | Yes |
| Is the sampling strategy relevant to address the research question? | Yes | Yes | Yes |
| Is the sample representative of the target population? | Yes | Yes | Yes |
| Are the measurements appropriate? | Yes | Yes | Yes |
| Is the risk of nonresponse bias low? | Can’t tell | Yes | Yes |
| Is the statistical analysis appropriate to answer the research question? | Yes | Yes | Yes |
| Is there an adequate rationale for using a mixed methods design to address the research question? | Yes | Yes | Yes |
| Are the different components of the study effectively integrated to answer the research question? | Yes | Yes | Yes |
| Are the outputs of the integration of qualitative and quantitative components adequately interpreted? | Yes | Yes | Yes |
| Are divergences and inconsistencies between quantitative and qualitative results adequately addressed? | Yes | Yes | Yes |
| Do the different components of the study adhere to the quality criteria of each tradition of the methods involved? | Yes | Yes | Yes |
| Percentage of criteria met (%) | 80 | 100 | 100 |
